# Adaptation and constraint in the evolution of the mammalian backbone

**DOI:** 10.1186/s12862-018-1282-2

**Published:** 2018-11-16

**Authors:** Katrina E. Jones, Lorena Benitez, Kenneth D. Angielczyk, Stephanie E. Pierce

**Affiliations:** 1 Museum of Comparative Zoology and Department of Organismic and Evolutionary Biology, Harvard University, 26 Oxford Street, Cambridge, MA 02138 USA; 2 Integrative Research Center, Field Museum of Natural History, 1400 South Lake Shore Drive, Chicago, IL 60605-2496 USA

**Keywords:** Vertebral column, Geometric morphometrics, Mammal evolution, Ecology, Locomotion, Adaptation

## Abstract

**Background:**

The axial skeleton consists of repeating units (vertebrae) that are integrated through their development and evolution. Unlike most tetrapods, vertebrae in the mammalian trunk are subdivided into distinct thoracic and lumbar modules, resulting in a system that is constrained in terms of count but highly variable in morphology. This study asks how thoracolumbar regionalization has impacted adaptation and evolvability across mammals. Using geometric morphometrics, we examine evolutionary patterns in five vertebral positions from diverse mammal species encompassing a broad range of locomotor ecologies. We quantitatively compare the effects of phylogenetic and allometric constraints, and ecological adaptation between regions, and examine their impact on evolvability (disparity and evolutionary rate) of serially-homologous vertebrae.

**Results:**

Although phylogenetic signal and allometry are evident throughout the trunk, the effect of locomotor ecology is partitioned between vertebral positions. Lumbar vertebral shape correlates most strongly with ecology, differentiating taxa based on their use of asymmetric gaits. Similarly, disparity and evolutionary rates are also elevated posteriorly, indicating a link between the lumbar region, locomotor adaptation, and evolvability.

**Conclusion:**

Vertebral regionalization in mammals has facilitated rapid evolution of the posterior trunk in response to selection for locomotion and static body support.

**Electronic supplementary material:**

The online version of this article (10.1186/s12862-018-1282-2) contains supplementary material, which is available to authorized users.

## Background

Disentangling the processes that lead to the evolution of complex biological structures is one of the major aims of evolutionary biology [1–4]. Integration between separate component parts of anatomical structures has been increasingly recognized as an important determinant of variation on genetic, phenotypic, and evolutionary scales [5–11]. Patterns of integration may enhance evolvability by compartmentalizing functional units into modules, limiting pleiotropic gene effects and thus promoting independent evolution in response to divergent selective regimes [4, 12–17]. The vertebrate axial skeleton represents the archetypal integrated structure because it is composed of repeating, serially-homologous vertebrae that have maintained remarkable self-similarity throughout vertebrate history [18, 19]. Simultaneously, the subdivision of the vertebral column into semi-autonomous modules (regions) has been achieved to varying degrees across different groups, with mammals constituting the best-known and most-extreme example [20–24]. Thus, the vertebrate axial skeleton provides a system with which to examine the interaction between integration, modularity, and morphological evolution over macroevolutionary timescales.

Evolution of the vertebral column is shaped not only by integration between vertebrae (serial homology), but also by phylogenetic and allometric constraints, as well as adaptation to diverse ecological niches. Phylogenetic history is a key predictor of vertebral variation, reflecting shared genetic, developmental, or functional interactions [25–28]. Allometry is also an important factor, as structural adaptation of vertebrae to static loading is necessary to accommodate increasing size [25, 27, 29–32]. The axial skeleton is integrally involved in body support and movement, respiration, and anchoring the head and limbs and, therefore, is subject to selection for a variety of functions and ecological specializations [22, 28, 33–35]. Further, evolutionary responses to these factors may be shaped by the modular organization of the vertebral column, resulting in serial modifications of morphological variation and disparity [24, 26, 30, 36].

Here we examine evolution of the mammalian ‘trunk’ (thoracic and lumbar vertebrae) to understand how regionalization of the vertebral column impacts macroevolutionary patterns. In most tetrapods, the vertebral column is relatively homogenous and vertebral counts vary widely between species. In mammals, however, the vertebral column is constrained in vertebral count but extremely variable in terms of vertebral morphology – resulting in discrete regions [37–40]. Most notably, the functional subdivision of the mammal trunk (thoracolumbar region) into respiratory (thoracic) and locomotor (lumbar) regions in the synapsid forerunners of mammals is a critical step in establishing the mammalian bauplan; demarcating the origin of novel vertebral functions and locomotor behaviors [20, 39, 41–44]. Whereas the thoracic region forms part of the ribcage, facilitating breathing and supporting the forelimb, the novel mammalian lumbar region is free of ribs and has been linked with various locomotor behaviors (e.g., hunting behavior [45], arboreality [46], posture [47, 48], running performance [49], jumping [50], swimming [33]). Therefore, the mammal trunk provides the ideal case study for understanding the implications of regionalization on vertebral variation, adaptation, and evolvability.

Our study asks how has thoracolumbar regionalization impacted adaptation and evolvability through mammalian evolution? To answer this question, we measured vertebral morphology in a wide range of extant mammals, spanning a variety of clades and sizes. We quantified the shape of five thoracolumbar vertebrae, selected to reflect variation among and between regions, using 3D geometric morphometrics and compared the influence of phylogeny, allometry, and ecology, as well as modularity, disparity and evolutionary rates along the column. Based on prior work, we predicted that phylogenetic history, allometry, and/or ecology would have significant effects on the overall morphology of the thoracolumbar region (Hypothesis 1), but that ecology would have greater influence in the lumbar region (Hypothesis 2). We also predicted that morphological variation would reflect the modular organization of the thoracolumbar region (Hypothesis 3), and if modularity and adaptation promote evolvability, that the lumbar region would display elevated disparity and evolutionary rates (Hypothesis 4).

## Methods

### Specimens and sample size

**Fig. 1:**
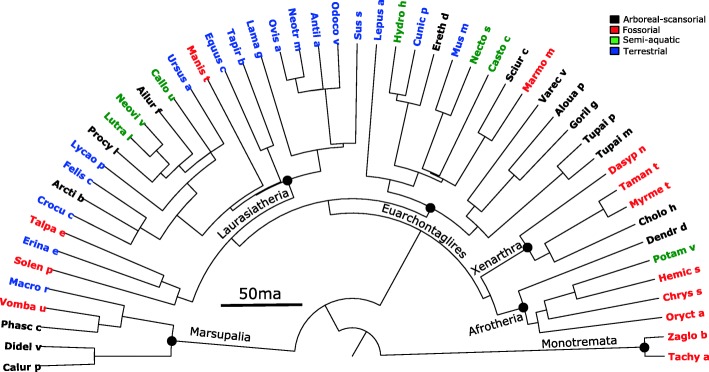
Time-calibrated phylogeny of the species included in this study. Based on a synthesis of relationships and branch lengths from a wide range of molecular studies [51]. Branch lengths scaled to time in millions of years. For full taxonomic names see Additional file 1: Table S6

Vertebrae from 52 mammalian species, including monotremes, metatherians, and representatives from all major eutherian clades, were selected to span mammalian phylogeny and to represent a broad diversity of locomotor ecologies (Fig. 1). Although some groups are relatively more abundant in collections (e.g., ungulates, carnivores), we attempted to sample evenly across the mammalian tree to avoid bias toward groups. Our sample size and selection of representatives from each major clade are comparable to a recent study that examined morphological divergence and evolvability in ray-finned fishes using similar methods [17]. A phylogeny for the mammalian taxa sampled was generated using timetree.org [51], which utilizes a hierarchical synthesis of published molecular timetrees to estimate topology and branch lengths (Fig. 1).

Specimens examined were primarily from the osteological collections of the Museum of Comparative Zoology (MCZ), Harvard University (Additional file 1: Table S5). Digital images of several specimens were also obtained from the online repositories ‘Morphosource’ and ‘Digimorph’, originally collected from the American Museum of Natural History (AMNH) and the Field Museum of Natural History (FMNH) (Additional file 1: Table S5). All specimens analyzed were adults (based on fusion of epiphyses), pathology-free, and with vertebrae accurately seriated (i.e. precise identification of the anterior-posterior position along the column).

This study utilized a broad phylogenetic sample to examine large-scale patterns across mammals. Therefore, we used very broad groupings to tease apart large-scale influences of ecology and applied multiple ecological assignments where appropriate. Information on locomotor ecology was gathered from the literature (see Additional file 1: Table S1 for literature sources), and species were classified into four categories according to the definitions used in previous studies [52]: terrestrial, scansorial-arboreal, fossorial and semi-aquatic. We combined scansorial and arboreal into a single group of climbing-specialized mammals to increase within-group sampling. Flying (e.g. bats) and fully aquatic (e.g. whales) taxa were not included due to their derived locomotor habits and the highly disparate selective pressures they likely encounter. A secondary locomotor classification was also defined for taxa which utilize multiple locomotor strategies based on behavioral descriptions or had multiple classifications in the literature (Additional file 1: Table S5).

To capture maximum shape variation along the vertebral column and compare taxa with varying vertebral counts, five thoracolumbar vertebrae per specimen were sampled, resulting in a total of 260 measured vertebrae. The vertebrae selected included: the first thoracic, the numerically mid-thoracic, the vertebra that marks the transition from horizontally- to vertically-oriented zygapophyses (diaphragmatic), the vertebra at one-third of lumbar length (anterior lumbar), and the final lumbar vertebra. As morphology varies strongly between vertebral regions, vertebrae were defined relative to regional landmarks (e.g., first free rib, diaphragmatic, last free rib) to enable identification of functionally homologous positions in taxa with varying vertebral formulas (Additional file 1: Figure S1). For a full description of the vertebrae selected for each species see Additional file 1: Supplemental Materials and Methods.

### Data acquisition and landmarks

**Fig. 2:**
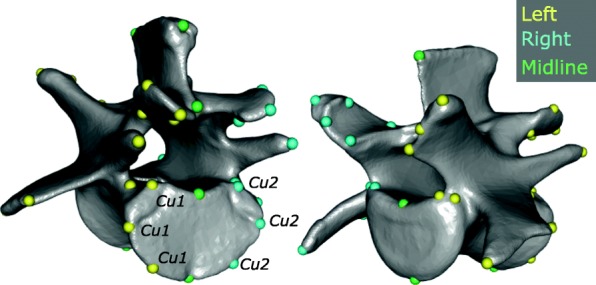
Three-dimensional landmarks collected on second lumbar vertebra of *Marmota monax.* For descriptions see Tables S1 and S2. Cu: curve

A three-dimensional geometric morphometric (GMM) approach was used to quantify the complex shape of the thoracolumbar vertebrae. For structures present throughout the column (centrum, arch, zygapophyses, neural spine) traditional homology-based landmarks were selected to capture shape variation (Fig. 2, Additional file 1: Table S1). The curvature of the endplate lacks suitable features for landmarking but is an important feature in determining articulation of the intervertebral joints. Therefore, sliding semi-landmarks were employed to capture the shape of the endplate [53]. Further, some muscular processes (anapophysis, metapophysis, transverse process) are variably present along the column, but represent a vital component of vertebral morphology. To include these structures, we adopted a redundant landmarking approach (following e.g. [54, 55]), which uses overlapping landmarks to record the presence or absence of serially-homologous structures along the column (Additional file 1: Table S2). For further details see Additional file 1: Supplementary Materials and Methods.

Large-bodied specimens (e.g., *Equus caballus*) were landmarked using a MicroScribe G2X Digitizer. For smaller specimens, landmarks were collected from three-dimensional models created using a Skyscan model 1173 micro-computed tomography (CT) scanner in the MCZ or from online repositories (see above). The target vertebrae were manually segmented and 3D rendered using Mimics software (Materialise, version 19) to make 3D surface meshes. Thirty-eight landmarks were placed manually using the MicroScribe or the ‘Measure and Analyze’ feature of the Simulate menu of Mimics. For the sliding semi-landmarks, two curves with greater than 10 landmarks each were collected along the left and right sides of the caudal endplate. The curves were later resampled to five landmarks each using the function *‘digit.curves’* in *geomorph* [56, 57]*.* The first and last landmarks of each curve were then replaced by single fixed dorsal and ventral midline centrum landmarks (Additional file 1: Table S1), ensuring symmetry when the semi-landmarks were slid between them. Thus, a total of 44 3D coordinates were collected for each vertebra (38 fixed landmarks and two curves with three sliding semi-landmarks each) (Fig. 2; see Additional file 1: Tables S1 and S2 for landmark descriptions). An error study was conducted on four species with four replicates, which demonstrated that landmarking accuracy was sufficient to distinguish both species and vertebrae using this protocol (see Additional file 1: Supplementary Material and Methods, Figure S2, Table S3).

### Data analysis

All analyses were conducted in R version 3.4.2 and the package *geomorph* version 3.0.5 [56, 57]. Landmark coordinates were aligned using generalized Procrustes superimposition (GPA) with the function ‘*gpagen*’ to remove size, rotation, and translation, and the semi-landmarks were permitted to slide to maximize bending energy. To remove asymmetry from the dataset, symmetrized Procrustes coordinates were generated using ‘*bilat.symmetry’*. Analyses were conducted on each of the five individual vertebral positions, separately, and on a ‘whole-column’ dataset (all vertebral positions combined per specimen) as described below.

### Individual vertebral positions

#### Shape variation

GPA and Principal components analysis (PCA) were used to visualize vertebral shape variation at the different vertebral positions – separately – using *‘plotTangentSpace’*. Shape variation at each vertebral position was visualized using mesh warping, based on the 3D surface mesh of the specimen lying closest to the mean shape for that vertebral position. Meshes were morphed to the mean shape for each position using ‘*mshape’* and *‘warpRefMesh’* and then to PCA extremes or ecological grouping means using ‘*plotRefToTarget’*.

#### Phylogeny, size and ecology

The multivariate K-statistic was used to estimate phylogenetic signal present in the symmetrized Procrustes coordinates [58, 59]. This was calculated using the function ‘*physignal*’ with 10,000 permutations to determine significance. Higher K-values correspond to a stronger phylogenetic signal, with values greater than one indicating that traits are conserved within the phylogeny, whereas values less than one indicate weaker phylogenetic signal and more convergence. To quantify the relationship between vertebral shape, size, and ecology, we used phylogenetically-corrected multivariate analysis of covariance (MANCOVA) in ‘*procD.pgls*’ [60, 61]. In this model, symmetrized Procrustes coordinates were the dependent variables with log centroid size (as a proxy for body size) as a covariate and ecological grouping (Additional file 1: Table S5) as a factor. As some taxa exhibit behaviors consistent with multiple ecological groups, the MANCOVA was run for both the primary and secondary ecological grouping assignments.

#### Modularity

Phylogenetically-corrected modularity was calculated as the CR coefficient, a ratio of the between-vertebra covariation to the within-vertebra covariation that reflects the relative independence of the vertebrae, using *‘phylo.modularity’* [62]. Pairwise variations in CR coefficient along the column were visualized using *‘corrplot’.* To ensure the dataset was appropriate for this analysis, stability of the covariance matrix for each vertebra was confirmed using random skewers analysis with 1000 bootstrap replicates (median *r* = 0.95) [63].

#### Disparity and evolutionary rate

Morphological disparity was quantified for each vertebral position using the ‘*morphol.disparity*’ function. Procrustes variances for each position were calculated, with log centroid size included as a covariate, and differences between vertebrae were tested using permutation [53]. Further, evolutionary rates at each vertebral position were calculated using *‘compare.multi.evol.rates’* [64], and the significance of between-position differences tested using phylogenetic simulation. Confidence intervals on disparity and rate measures were calculated based on randomized residuals from a linear model using *‘procD.lm’* and *‘procD.pgls’*.

### Whole-column analysis

#### Shape, phylogeny, and ecology

The vertebral column, though composed of individual vertebrae, functions as a unit and is developmentally and evolutionarily integrated [22]. Therefore, in addition to examining the evolution of individual vertebrae, it is also important to consider evolutionary influences on the whole thoracolumbar column. To examine the evolution of multiple vertebrae simultaneously, we conducted a combined analysis of all five vertebral positions. Procrustes-aligned landmarks (single fit for all vertebrae) from each of the five vertebral positions for each species were concatenated to produce a new ‘whole-column’ coordinate set. To reduce the dimensionality of the dataset, only midline and left-side landmarks were used, resulting in a total of 130 landmarks for each specimen, which is within the dimensionality range of other comparative morphometric studies [65–67]. This ‘whole-column’ landmark set was then subjected to the shape, phylogenetic signal, and MANCOVA analyses described above to examine the influence of phylogeny, size, and ecology on total thoracolumbar evolution. The residual randomization tests employed by ‘*ProcD.pgls*’ are specifically designed for, and are robust to, high dimensionality data [60, 68]. As each vertebra was treated separately in the initial Procrustes superimposition, the relative contribution of each vertebral position to the whole-column was examined by summing the absolute PC loadings of each landmark across the individual vertebral position. Whole-column PC axes reflect shape variation in all five vertebral positions simultaneously. Therefore, shape variation along PC axes was visualized by extracting loadings for each individual vertebral positions from the concatenated PC loadings, and warping the vertebrae as described above.

#### Method validation

Previous studies quantifying the shape of multiple elements simultaneously have either combined PC or relative warps scores from separate analyses to produce new shape variables [27, 69, 70]. Due to the common landmarking approach for each vertebra taken here, we were able concatenate Procrustes-coordinates directly, producing a mathematically-identical shape space to that obtained based on PC concatenation. To validate this approach, we simulated five hypothetical vertebral columns with serial variation based on rectangles (Additional file 1: Supplementary materials and methods). This analysis demonstrated that a morphospace based on Procrustes concatenation was better able to distinguish unique column morphologies than the traditional approach of plotting all vertebrae as independent elements (Additional file 1: Figures S3, S4), and that our approach produces identical results to concatenation of principal component scores (Additional file 1: Figures S4, S5, Table S4).

## Results

### Whole-column shape

**Fig. 3:**
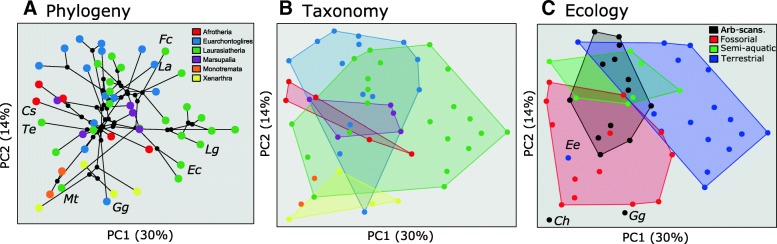
Whole-column analysis of vertebral shape. PCA based on concatenated ‘whole-column’ shape (see Methods, Additional file 1: Supplementary Materials and Methods). A. Phylogenetic relationships plotted to create a phylomorphospace. *Cs: Chyrsochloris stuhlmanni; Te: Talpa europaea; Mt: Manis temminckii; Gg: Gorilla gorilla; Ec: Equus caballus; Lg: Lama glama; La: Lepus americanus; Fc: Felis catus.* B. grouped by Superorder; C. grouped by locomotor ecology. *Ch: Choloepus hofmanni; Ee: Erinaceus europaeus; Gg: Gorilla gorilla*

The thoracolumbar column functions as an integrated unit, therefore it is important to consider variation in total thoracolumbar morphology. Combining Procrustes coordinates across five vertebral positions into a whole-column morphospace enables taxonomic and ecological groups to be distinguished (Fig. 3b and c; Additional file 1: Supplemental Materials and Methods). Landmarks from all the vertebral positions contribute to whole-column variation in the top three PCAs (those contributing > 5% variance, 51% total variance), highlighting the importance of extracting common patterns of variation along the column (Table 1).

**Table 1:** Summed loadings of landmarks from each vertebra on the whole-column PCA

	Tot Var.	T1	Mid-T	Dia	Ant L	Last L
PC1	30.0%	22.0%	23.2%	16.1%	20.6%	18.2%
PC2	13.5%	17.2%	9.6%	17.2%	29.6%	26.4%
PC3	7.5%	25.9%	23.5%	20.2%	16.3%	14.1%

*Tot var.* Total variation explained by that PC, *T1* First thoracic, *Mid-T* mid-thoracic, *Dia* diaphragmatic, *Ant L* anterior lumbar, *Last L* last lumbar

Although Principal component axes from the whole-column PCA represent concurrent variation in all five vertebrae simultaneously, associated shape variation for each underlying vertebra was visualized by extracting the individual position loadings from the concatenated shape (Additional file 1: Figure S6). PC1 reflects variation from tall thoracics with large neural spines, and wide lumbars with elongate transverse processes (positive scores); to vertebrae with relatively smaller muscular processes throughout the column but larger metapophyses (negative scores) (Additional file 1: Figure S6). Positives scores on PC2 correspond to columns with wide-set (cervical-like) zygapophyses on T1, strongly anticlinal (varying from caudally to cranially directed) neural spines, and ventrally-inclined lumbar transverse process, typified by most euarchontoglires and some laurasiatheres (Additional file 1: Figure S6). Negative scores on PC2 represent columns with narrow-set (thoracic-like) zygapophyses on T1, caudally-directed neural spines throughout the column, and short, perpendicular lumbar transverse processes.

There are significant effects of phylogeny, size, and ecology on whole-column shape, corroborating Hypothesis 1 (Table 2). Although members of some clades cluster in the whole-column morphospace (Fig. 3b), the relatively low K value of 0.63 indicates deviation from pure Brownian motion (Table 2). Further, the distribution of taxa suggests considerable homoplasy, with certain clades (e.g., laurasiatheres) invading multiple regions of morphospace (Fig. 3a, b).

**Table 2:** Effects of phylogeny, size, and ecology on whole-column morphology and individual vertebral positions

Vertebral Position	Phylogeny		Allometry		Ecology		Ecology2	
	K	*p*-val.	Rsq	*p*-val.	Rsq	*p*-val.	Rsq	*p*-val.
Whole Column	**0.63**	**0.001**	**0.12**	**< 0.001**	**0.09**	**0.012**	**0.08**	**0.032**
First Thoracic	**0.65**	**0.001**	**0.16**	**< 0.001**	0.07	0.112	0.07	0.174
Mid Thoracic	**0.72**	**0.001**	**0.14**	**< 0.001**	0.08	0.086	0.08	0.066
Diaphragmatic	**0.54**	**0.001**	**0.10**	**< 0.001**	0.05	0.535	0.06	0.331
Second Lumbar	**0.63**	**0.001**	**0.13**	**< 0.001**	**0.11**	**0.006**	0.08	0.051
Final Lumbar	**0.66**	**0.001**	**0.10**	**< 0.001**	**0.13**	**0.001**	**0.09**	**0.048**

Phylogenetic signal was measured using the K-statistic. Allometry, ecology, and their interaction from a phylogenetic MANCOVA. *Rsq* R-squared value. *P-val. p* value, *Ecology2* secondary ecology assignment used

Bold values indicate significance at the 0.05 alpha level

Post-hoc comparisons of ecological groups reveal significant differences in whole-column shape between terrestrial and fossorial, and to a lesser extent, scansorial-arboreal species (*p* = 0.028, *p* = 0.056). Taxa utilizing fossorial or scansorial-arboreal habits tended to have low PC1 scores, reflecting relatively small to absent (in the case of monotremes) lumbar transverse processes and small neural processes, but extremely large metapophyses (Fig. 3, Additional file 1: Figure S6, S7). Terrestrial taxa have high PC1 scores indicating larger neural spines and transverse processes. Repeated invasion of low PC1 regions of morphospace indicates convergent evolution of this morphology several times within Mammalia in association with digging or climbing ecologies. For example, the highly fossorial taxa *Talpa europaea* and *Chrysochloris stuhlmanni,* (Fig. 3a: Te, Cs) lie on the far negative PC1, despite being members of distantly-related clades. Similarly, the pangolin *Manis temminckii* (Fig. 3a: Mt) lies at negative PC1 near other fossorial taxa (e.g., monotremes), and far from its sister group the Carnivora.

Among the terrestrial taxa, PC2 appears to distinguish dorsostable ungulates (e.g., *Equus caballus, Llama glama;* Fig. 3a: Ec, Lg) from dorsomobile runners (e.g., *Felis catus, Lepus americanus;* Fig. 3a: Fc, La). This variation reflects elongate and obliquely oriented transverse processes and cranially-inclined neural spines in the dorsomobile species (high PC2), and long but perpendicularly oriented transverse processes in the dorsostable species (low PC2) (Additional file 1: Figure S6).

*Gorilla gorilla* and *Choloepus hoffmanni* are outliers from the arboreal group, with relatively negative PC1 scores, likely relating to their suspensory mode of locomotion (Fig. 3c: Ch, Gg). Similarly, the hedgehog *Erinaceus europaeus* is also an outlier from the terrestrial group, likely reflecting phylogenetic inertia as it clustered with other ecologically-disparate eulipotyphlans (Fig. 3c: Ee), instead of species with more similar ecologies.

### Individual vertebral shape

**Fig. 4:**
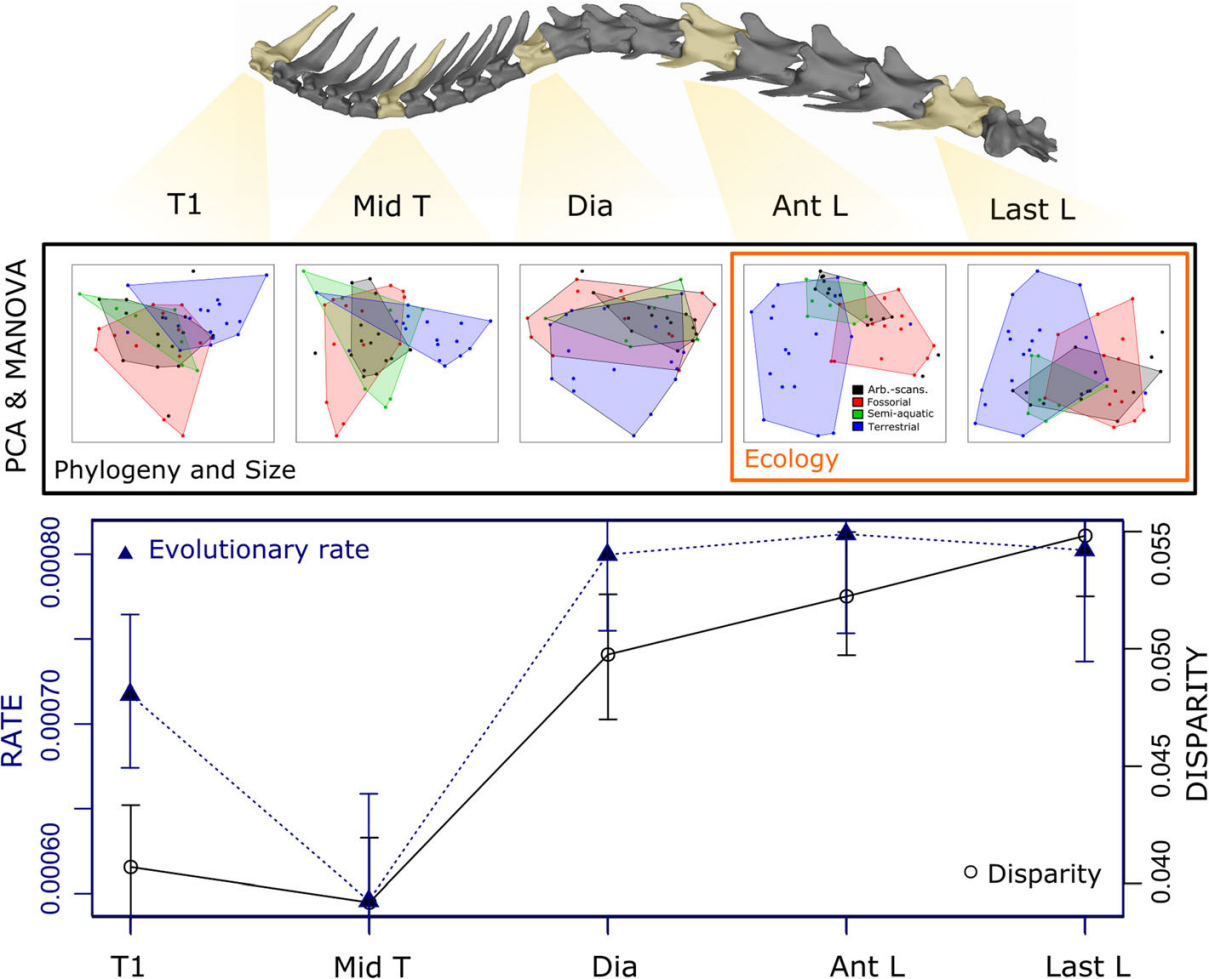
Along-column variation in morphology, disparity and rate. PCA’s representing morphological variation at each vertebral position. Black box: significant effect of phylogeny and size; Orange box: significant effect of locomotor ecology. Disparity (Procrustes variance) and evolutionary rate at each position shown below, with confidence intervals based on randomized residuals. T1: First thoracic; Mid-T: mid-thoracic; Dia: diaphragmatic; Ant L: anterior lumbar; Last L: last lumbar

A more complex story is revealed when evolutionary factors are broken down by vertebral position. Although phylogeny and size have a significant effect at all five vertebral positions, ecology has a significant effect only at the last two positions (anterior lumbar and last lumbar) (Table 2, Fig. 4). This finding corroborates Hypothesis 2 in suggesting enhanced ecological influences in the posterior column. Post-hoc pairwise tests indicate significant differences between terrestrial and scansorial-arboreal-fossorial taxa in the lumbar region (Anterior: P_Arb-Ter_ = 0.030, P_Foss-Terr=_0.033; Last: P_Arb-Terr_ = 0.034, P_Foss-Terr=_0.024) are driving this pattern. In PCA space, the terrestrial group (blue) has more negative PC1 scores than scansorial-arboreal (red) and fossorial (black) groups at the last two vertebral positions (Fig. 4). Terrestrial taxa (negative PC1) are typified by longer neural spines and transverse processes, which are cranio-ventrally deflected; whereas arboreal and fossorial taxa are characterized by short processes and large metapophyses (Additional file 1: Figure S7).

### Modularity

**Fig. 5:**
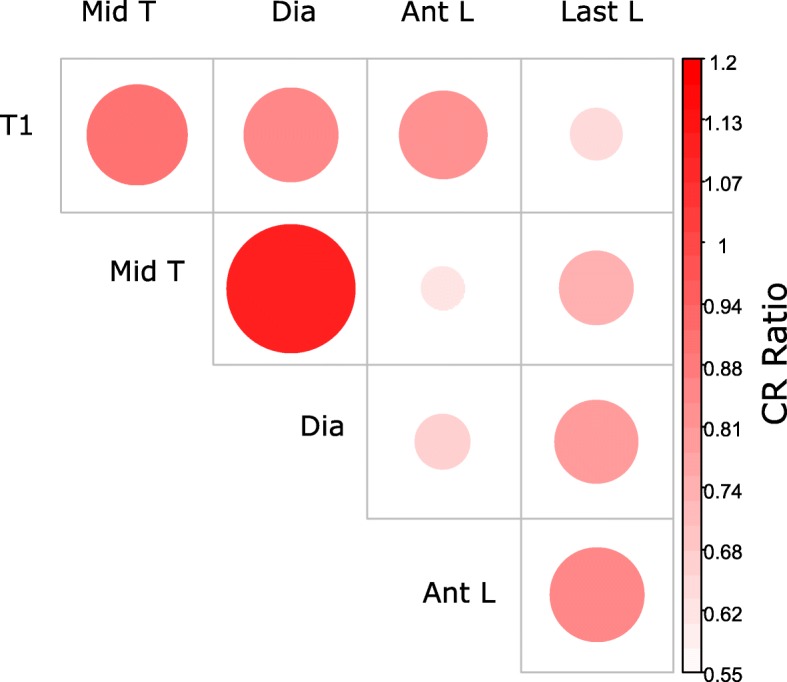
Modularity. Modularity measured as the CR ratio: a ratio of between-vertebra to within-vertebra covariation. Small, lightly colored circles indicate greater modularity (independence between vertebral positions), while large, darkly colored circles indicate greater integration (covariation between vertebral positions)

Trunk vertebrae are significantly modular, both with (CR = 0.798, *p* = 0.001) and without (CR = 0.748, *p* = 0.001) phylogenetic correction, indicating that despite strong covariation along the column vertebrae can still evolve somewhat independently. The CR ratios are below one, suggesting relative independence between vertebral positions. While between-vertebra modularity based on raw data reflects a simple relationship with vertebral position (closer vertebrae more integrated), phylogenetic modularity reflects are more complex pattern (Fig. 5a). The first three positions (T1 to diaphragmatic) and last two positions (anterior and last lumbar) have higher CR values, and thus are less independent than any other combinations. This supports the prediction that thoracic vertebrae and lumbar vertebrae form semi-independent modules (Hypothesis 3).

### Disparity and evolutionary rate

Disparity and evolutionary rate both increase posteriorly along the thoracolumbar column. Pairwise comparisons reveal that the lumbar positions have significantly higher Procrustes variance than the first two thoracic positions (Table 3, Fig. 4), corroborating Hypothesis 4. The diaphragmatic vertebra exhibits intermediate levels of disparity. Similarly, rates of evolution are also greatest in the posterior trunk, with the last three positions exhibiting significantly higher rates than the first or mid- thoracic positions (Table 3, Fig. 4).

**Table 3:** Pairwise *p*-values for between-position differences in disparity (upper) and evolutionary rate (lower)

		Disparity				
		T1	Mid-T	Dia	Ant L	Last L
Evo. Rate	T1	1.000	0.768	0.084	**0.029**	**0.008**
	Mid-T	**0.001**	1.000	0.051	**0.014**	**0.002**
	Dia	**0.025**	**0.001**	1.000	0.638	0.327
	Ant L	**0.012**	**0.001**	0.761	1.000	0.621
	Last L	**0.022**	**0.001**	0.931	0.807	1.000

*T1* First thoracic, *Mid-T* mid-thoracic, *Dia* diaphragmatic, *Ant L* anterior lumbar, *Last L* last lumbar

Bold values indicate significance at the 0.05 alpha level

## Discussion

Using geometric morphometrics, we quantified morphological variation in serially-homologous vertebrae to examine how vertebral regionalization has impacted adaptation and evolvability of the mammalian thoracolumbar column. Although the effects of phylogeny and size appear to be consistently important throughout the mammalian trunk, the evolutionary response of vertebral morphology to ecology is partitioned between vertebral positions, implicating vertebral regionalization as an important mechanism facilitating adaptation of the vertebral column to divergent ecological and locomotor functions.

### Phylogeny and size constraints

Similarity due to shared common ancestry (‘phylogeny’) is a major factor driving the patterns of morphological variation among species. The results presented here support previous work demonstrating the importance of phylogeny to vertebral variation [25, 27, 31]. Recovered K-values ranged between 0.55 and 0.65, indicating that although the effect of phylogeny was significant in shaping mammalian vertebral morphology, the variation in structure differed from that expected under the null hypothesis of Brownian motion [58, 59]. Variation in size places structural constraints on the axial skeleton due to its role in resisting gravitational loads [28, 30, 71], resulting in allometry in axial structure and function within groups (e.g., felids [31, 72, 73], bovids [25, 32], and kangaroos [27]). We further confirm this finding on a broader evolutionary scale by showing a significant influence of size on both the whole-column and individual vertebral positions in our cross-mammalian sample (Table 2).

### Ecological effects

Mammals employ diverse locomotor strategies to exploit their environment, which is often reflected in their postcranial anatomy [52, 65, 74–78]. Our results indicate a significant effect of locomotor ecology in the thoracolumbar column, even after phylogenetic and size correction (Table 2). The importance of the axial system in locomotion is highlighted by the major patterns of functional variation recovered in this analysis. The most striking trend in both the whole-column analysis (Fig. 3), and in the lumbar vertebrae (Fig. 4), was the contrast between terrestrial and fossorial/scansorial-arboreal morphologies. Interestingly, scansorial-arboreal and fossorial taxa partially overlapped in morphospace. This may reflect some overlap between these behaviors in certain taxa (e.g., *Tamandua tetradactyla, Erithizon dorsatum, Manis temminckii*), or similar functional demands placed on the axial skeleton by these seemingly disparate ecologies.

We hypothesize that the morphological patterns detected here reflect the relative importance of asymmetric (running) gaits in terrestrial taxa over symmetric (slower) gaits in fossorial/scansorial-arboreal species. Terrestrial taxa were typified by high PC1 scores in the whole column analysis, indicating elongated neural spines and transverse processes, whereas fossorial/scansorial-arboreal taxa tended to have lower PC1 scores consistent with well-developed metapophyses (Additional file 1: Figure S6). Enlarged metapophyses provide insertions for *mm. transversospinalis* and deep portions of *M. longissimus dorsi* in therians [28], whereas *M. longissimus dorsi* is almost entirely absent in monotremes [79]*.* Therefore, the enlarged metapophyses in fossorial and some scansorial-arboreal groups may reflect enhanced stabilization of the trunk against strong limb motions during digging or climbing via *mm*. *transversospinalis* (e.g., *m. multifidus*) and deeper insertions of *M. longissimus* [28]*,* as well as restricted use of asymmetric gaits (which require an alternate arrangement of axial musculature) during non-terrestrial locomotion in these taxa.

Kinematic and anatomical data support this hypothesis. Comparative anatomical analyses of opossums, fossorial armadillos, and suspensory-arboreal sloths demonstrated significant increases in the size of *mm. transversospinalis* and *m. iliocostalis* in the fossorial and arboreal species, with a relative reduction of *M. longissimus* [80, 81]*.* Likewise, kinematic data from the sloth (*Choloepus didactylus*) show both the absence of asymmetric gait-use, and reduction of sagittal bending in favor of lateral and torsional motion during arboreal locomotion relative to typical mammalian gaits [82]. Together, these data suggest that enlargement of *m. transversospinalis* and *m. iliocostalis* stabilizes the trunk against sagittal and lateral forces generated during digging and climbing behaviors, and that reduction of *M. longissimus* reflects the lack of asymmetric gaits that depend upon sagittal motions [80–82]. This suspensory-arboreal locomotor specialty is reflected in the divergent vertebral morphology of the sloth examined in this study, which lies at the minimum of both PC1 and PC2. Our data provide osteological evidence that the link between epaxial myology and behavior may be widespread among mammals, and bony features (e.g., enlarged metapophysis relative to neural spine and transverse processes) may be useful for identifying fossorial and arboreal behaviors in the fossil record.

### Locomotor adaptation and evolvability in the lumbar region

Although there was a significant influence of phylogeny and size throughout the trunk, variation associated with locomotor ecology was focused in the lumbar region (Table 2). The strong correlation of ecology and lumbar morphology reflects the important role of the lumbar region in mammalian locomotion. Whereas most tetrapods emphasize lateral bending of the trunk during locomotion, mammals employ sagittal bending during asymmetric gaits (e.g., galloping, bounding) or during leaping and jumping [50, 83, 84]. This specialized mobility of the spine has been posited to be associated with the evolution of a ribless lumbar region because sagittal motions are restricted to the posterior trunk (though they are not perfectly correlated with the thoracolumbar transition), and the vertical orientation of the zygapophyses in this region is thought to facilitate sagittal bending [83, 85]. In addition to the strong effect of ecology, there is also an increase in morphological disparity and evolutionary rates in the posterior trunk (Fig. 4). These results suggest natural selection for locomotor efficacy may be playing a role in driving rapid morphological evolution in the lumbar region, resulting in the acquisition of new, diverse morphologies.

A link between lumbar shape, locomotor ecology, and disparity has been proposed at a smaller phylogenetic scale within Felidae (cats) [31, 45]. However, unlike the felid dataset, which indicated significant ecological signal in the lumbar and diaphragmatic (transitional) regions, we did not find a significant correlation at the diaphragmatic position (Table 2, Fig. 4). Therefore, we hypothesize that the posterior thoracic vertebrae may be variably recruited into locomotor function with the lumbar region between taxa. This idea is supported by kinematic data showing that the cranial extent of sagittal bending during running varies, including posterior thoracics in some species but restricted to the lumbar region in others [83]. Due to their highly dorsomobile gait, felids likely involve the diaphragmatic region during running, resulting in the correlation between locomotor ecology and diaphragmatic morphology [86, 87].

### Gradational selection and modularity in the mammal trunk

**Fig. 6:**
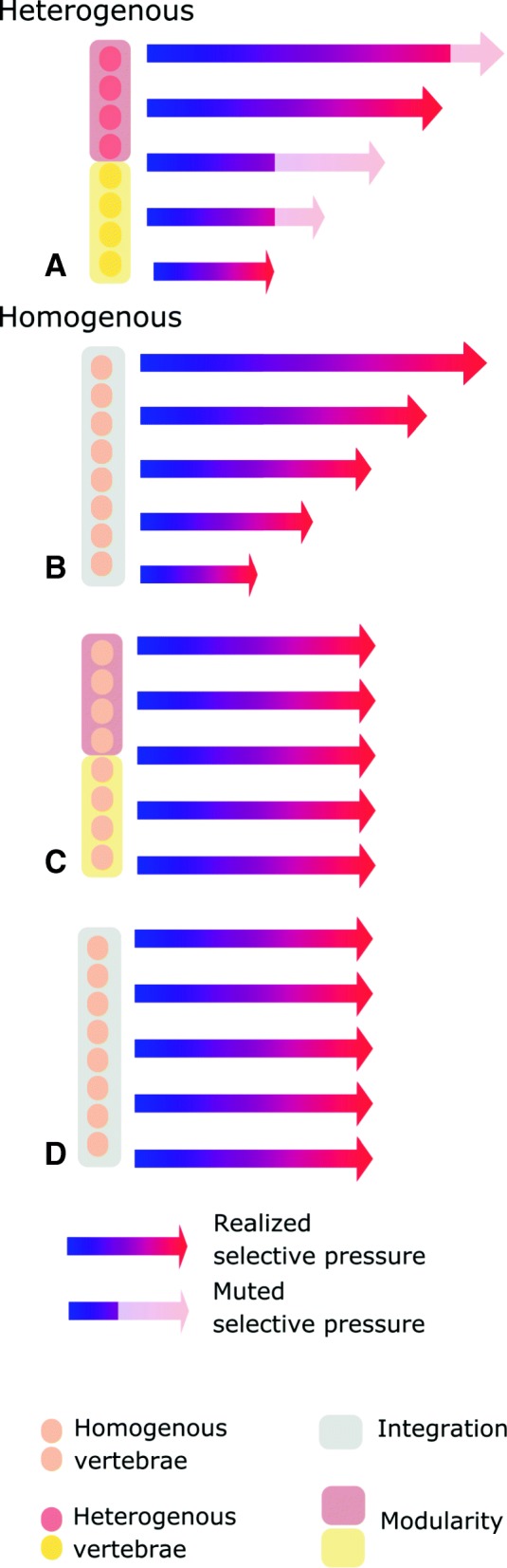
Hypothesized relationship of selection and modularity in the axial skeleton. The strength of selection may vary along the vertebral column (length of arrows, **a**, **c**), but the evolutionary response to that selection is modulated by integration patterns (shading on arrows). Four different evolutionary scenarios are hypothesized. **a** Modularity and gradational selection. Selection varies along the column and the selective regimes of different modules may diverge (stronger in the red zone than the yellow zone) due to limited integration between red and yellow modules. Such a scenario is proposed to explain the heterogenous vertebral column of mammals. Homogeneous vertebral columns may result from either increased integration, uniform selection or both. **b** Integration and gradational selection. Selection regimes vary along the column, but morphological variation is muted by strong integration between vertebrae. **c** Modularity with uniform selection. Although the potential for generating evolutionary variation between vertebrae exists, selection maintains uniformity along the column. **d** Integration with uniform selection. Both factors limit craniocaudal variation. Circles: vertebrae; shaded boxes (yellow, red, grey): modules; colored arrows: selective pressure; greyed-out arrows: selective pressure muted by serial integration

Relative to most other tetrapods, the mammalian vertebral column is strongly differentiated into regions with distinct morphologies. Two essential components are required to explain this heterogeneity: disparate selective regimes and modularity. Disparate selective regimes may be generated by selection gradients formed along the vertebral column. Due to its anatomical connection between the forelimb and hind limb, and its elongate structure, biomechanical forces in the trunk (and the vertebral column in particular) act in a strongly gradational manner. For example, mediolateral forces generated by the pelvis and hind limb during locomotion form a decreasing force gradient – from high caudally to low cranially [88]. Likewise, the ventral ‘sagging’ forces placed upon the trunk due to support of body mass between the limbs gradually reach a peak at the mid-trunk [28, 89], and sagittal bending contributing to pelvic displacement during mammalian asymmetric gaits increases posteriorly along the trunk [83]. These forces may generate highly heterogenous selection regimes for vertebrae at different positions, such that the selection pressures acting upon each vertebra may form a gradient (Fig. 6a, arrows), providing impetus for divergent evolution along the column.

Serially-homologous structures tend to covary strongly due to shared developmental origins, which can act as a developmental constraint on intracolumn variation [14, 90]. However, modularity, and the subdivision of the column into regions, provides a mechanism for limiting or modulating this constraint. In *Mus*, anteroposterior expression of *Hox* genes correlate with craniocaudal region boundaries in adult morphology, implicating these genes in controlling vertebral regionalization [91, 92]. Further, knock-out experiments suggest that *Hox10* is crucial for patterning the ribless lumbar region in mammals [93]. This underlying developmental modularity is reflected by the evolutionary phenotypic modularity across the broad range of mammal taxa examined here, (Fig. 5, [23]), providing a mechanism for partitioned evolutionary responses to gradational selection (Fig. 6a).

Compared to mammals, the extinct forerunners of mammals, the non-mammalian synapsids, exhibit relatively homogeneous vertebral columns with little regional differentiation [39, 44, 94]. This suggests stronger integration between vertebrae (Fig. 6b), more uniform selection (Fig. 6c), or a combination of these factors (Fig. 6d). Recent analyses of subtle gradients in vertebral morphology suggest that basal synapsids had fewer vertebral regions than crown therians, indicating that they may also have been less modular [44]. The appearance of the ribless lumbar region in Mesozoic mammals has been used to infer shifts in *Hox* function that could signal increasing vertebral modularity in mammalian evolution [95]. Although the evolution of vertebral function in fossil synapsids is poorly understood, basal members of the group likely lacked asymmetric gaits and sagittal vertebral flexion [39, 96]. Therefore, therian mammals are derived in terms of both modularity and function, and either or both factors may play a role in the evolution of vertebral heterogeneity. Modularity itself may arise either by ‘variational adaptation’ – in which the evolution of reduced covariation between regions is a direct response to divergent selective regimes – or from the indirect erosion of developmental-genetic interactions between regions in response to selection for phenotypic robustness against pleiotropy [14]. Further work examining vertebral patterning and the relationship between regionalization and vertebral function in synapsids is required to tease apart this complex issue.

The data presented here suggest that locomotor selection for sagittal bending acting on the posterior trunk in mammals exploits modular variation to enable divergent evolution of vertebral regions. Modularity may lead to increased population-level and macro- evolutionary rates by aligning the genetic variation with the direction of selection, and limiting the interference between regions adapted for different functions [4, 15]. Thus, the serial morphology of the mammalian vertebral column reflects both the gradational selection and the integration patterns imposed by its modular structure.

## Conclusions

Relative to other tetrapods, mammals have highly differentiated thoracic and lumbar trunk regions. Here we demonstrate that modularity of the thoracolumbar column in mammals is accompanied by divergence of evolutionary responses in serially homologous vertebrae. Overall, vertebral shape is influenced by phylogeny, size and ecological specialization. However, the important role of the mammalian lumbar region in locomotion is reflected in enhanced ecological adaptation. More significantly, our data show that elevated adaptability of the posterior trunk across mammals is linked with increased disparity and evolutionary rates. For example, evolution of fossorial and arboreal behaviors across the mammal tree resulted in repeated convergence of lumbar morphologies linked with the loss of asymmetric gaits. Strong gradational selection for behaviors such as these, coupled with modular variation, likely facilitated rapid and disparate evolution of the lumbar region in mammals.

## Additional file

Additional file 1: Supplementary Materials and Methods, Figures S1-S7, Tables S1-S5, Supplementary references. (PDF 753 kb)

## Abbreviations

AMNH: American Museum of Natural History
CT: Computed tomography
FMNH: Field Museum of Natural History
GMM: Geometric morphometrics
GPA: Generalized Procrustes superimposition
MANCOVA: Multivariate analysis of covariance
MCZ: Museum of Comparative Zoology
PCA: Principal components analysis
T1: First thoracic
