# Supplementary material for: Adaptation and constraint in the evolution of the mammalian backbone
Source: BMC Evol Biol. 2018 Nov 16;18:172. doi: 10.1186/s12862-018-1282-2 (PMC6240174; doi:10.1186/s12862-018-1282-2)
Supplement: Supplementary file 1 — Supplementary Materials and Methods, Figures S1-S7, Tables S1-S5, Supplementary references. (PDF 753 kb) [file 12862_2018_1282_MOESM1_ESM.pdf]

SUPPLEMENTARY MATERIAL FOR:

ADAPTATION AND CONSTRAINT IN THE EVOLUTION OF THE MAMMALIAN BACKBONE

K. E. Jones, Benitez, L. , K. D. Angielczyk, S. E. Pierce

SUPPLEMENTARY MATERIALS AND METHODS

ADDITIONAL TABLES

ADDITIONAL FIGURES

SUPPLEMENTARY REFERENCES

SUPPLEMENTARY MATERIALS AND METHODS

Selecting vertebrae for analysis

Vertebrae were selected to capture the maximum range of morphological variation along the column. To assist with selecting appropriate vertebrae, patterns of morphological variation were measured in *Felis catus* using 19 linear and angular measures. The variation in these measures was summarized using a distance-based ordination (Principal Coordinates Analysis) based on Gower distances. Though variation along the thoracolumbar region is gradational, morphological differences are greatest between the anterior thoracic region (prediaphragmatic) and lumbar region, with the diaphragmatic vertebra forming a transition point between these two extremes (Figure S1). We therefore sampled five vertebrae: the diaphragmatic vertebra, which forms the transition between anterior and posterior column, two vertebrae anteriorly, and two posteriorly.

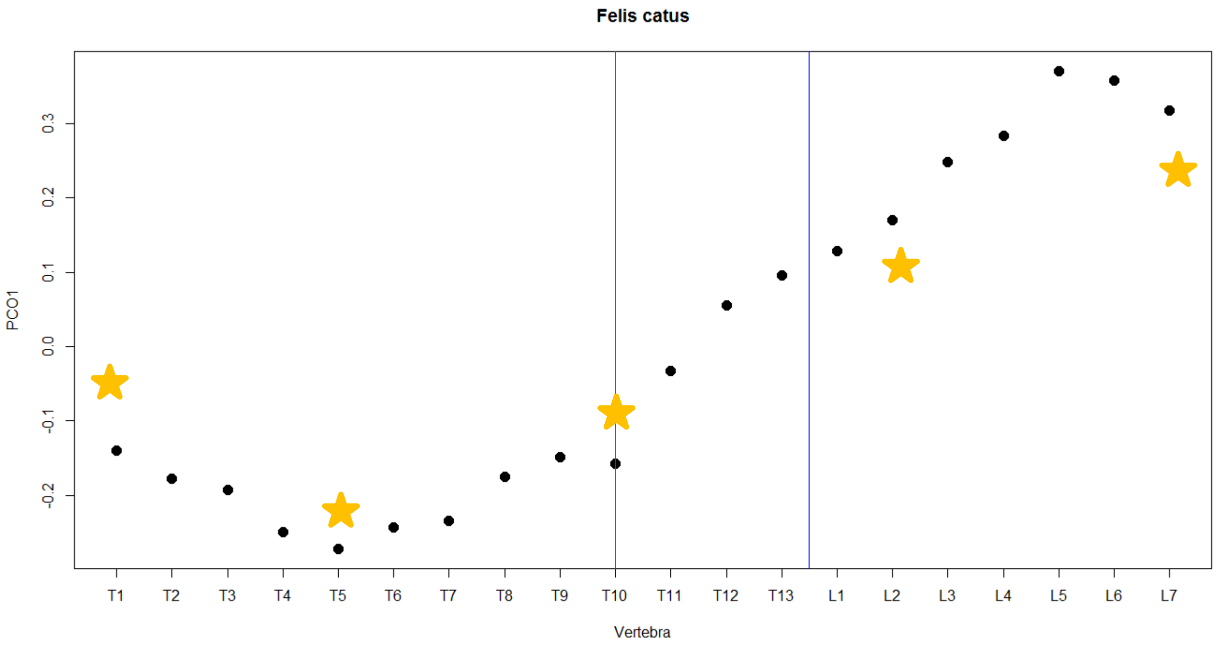

Figure S1: Morphological variation in *Felis catus*.
Characterized by Principal Coordinates Analysis of linear and angular measures. Red line: Diaphragmatic vertebra, blue line: thoracolumbar transition. Stars: Vertebrae selected for study.

Selecting vertebrae across varying counts – Thoracolumbar formula varies across mammals; therefore, absolute vertebral position (e.g., % length along column) provides a poor measure of functional homology between species. The vertebral column is subdivided into morphological regions that share developmental patterning (through *Hox* genes) and functional capabilities. For example, the thoracic region bears mobile ribs and forms the respiratory cage, whereas the lumbar region lacks them. Similarly, the diaphragmatic vertebra separates anterior

vertebrae with horizontal zygapophyses and posterior vertebrae with vertical zygapophyses, a difference that has been linked with vertebral function. To select functionally homologous vertebrae across columns with varying vertebral counts, we defined a sampling strategy relative to these key functional landmarks in the thoracolumbar column. We selected five vertebrae as follows, values in square brackets refer to the cat example provided above (Figure S1):

1. First thoracic – the most anterior vertebra bearing facets for the articulation of ribs, marking the anterior border of the thoracolumbar region [T1]
2. Mid-thoracic – the numerically middle vertebra between the diaphragmatic vertebra (which marks the transition to the posterior column) and the first thoracic. [T5]
3. Diaphragmatic – the vertebra marking the transition from horizontally-oriented to vertically-oriented zygapophyses (e.g., *Felis catus*, above). Where this transition is gradual (e.g., *Didelphis virginianus*), the most anterior vertebra was selected. [T10]
4. Anterior lumbar – the vertebra numerically one-third of the way along the lumbar region, usually L2 when lumbar counts are between 5 and 7. This vertebra was selected because the first lumbar is often very transitional in morphology and may lack well-developed transverse processes. [L2]
5. Last lumbar – the final lumbar which articulates directly with the sacrum. [L7]

Selecting landmarks

Landmarks were selected to fully capture all aspects of the shape of the vertebra. Although some of these features are invariant in their presence across the sampled vertebrae and taxa, some are variably present and therefore must be accounted for in the digitizing scheme.

Invariant features – Invariant vertebral features include the centrum, arch, zygapophyses and neural spine. We captured these features using eight midline landmarks, ten bilateral landmarks, and one bilateral curve consisting of three sliding landmarks.

Table S1: Invariant landmarks

	**Midline Landmarks**	
1	Cranial endplate - ventral at midline	*Ventral-most extent of endplate*
2	Cranial endplate - dorsal at midline	*Dorsal-most extent of endplate*
3	Cranial lamina at midline	*Cranial-most extent of neural lamina*
4	Cranial tip of neural spine	*Dorso-cranial tip*
5	Caudal tip of neural spine	*Dorso-caudal tip*
6	Caudal lamina at midline	*Caudal-most extent of neural lamina*
7	Caudal endplate – dorsal at midline+	*Dorsal-most extent of endplate*
8	Caudal endplate – ventral at midline+	*Ventral-most extent of endplate*
	**Bilateral Landmarks**	
9	Cranial endplate-arch - dorsal	*Base of neural arch on medial aspect*
10	Cranial endplate-arch - lateral	*Base of neural arch on lateral aspect*
11	Caudal arch base	*Cranial-most point*
12	Pre-zygapophysis - cranial extent	*Margin of zygapophyseal facet*

13	Pre-zygapophysis - caudal extent	*Margin of zygapophyseal facet*
14	Pre-zygapophysis – medial/ventral extent*	*Margin of zygapophyseal facet*
15	Pre-zygapophysis – lateral/dorsal extent*	*Margin of zygapophyseal facet*
16	Post-zygapophysis - cranial extent	*Margin of zygapophyseal facet*
17	Post-zygapophysis - caudal extent	*Margin of zygapophyseal facet*
18	Post-zygapophysis – medial/ventral extent*	*Margin of zygapophyseal facet*
19	Post-zygapophysis – lateral/dorsal extent*	*Margin of zygapophyseal facet*
	**Semi-landmarks**	
Curve	Caudal endplate outline – 3 landmarks	*Lateral boundary of endplate excluding hemi-facets. Starts and ends at midline L7/8.*

*In postdiaphragmatic vertebrae, the facet is rotated such that the medial border is more ventral, and the lateral border is more dorsal.
+Fixed landmarks defining beginning and end of bilateral curves

Variant features- Several other functionally importance aspects of vertebral morphology are highly variable between loci and taxa, and thus are more challenging to characterize using geometric morphometrics. Specifically, the metapophysis (or mammillary process), anapophysis (accessory process), and transverse process are variably present in this sample.

To overcome this challenge, we apply the 'degenerate' landmarking approach advocated by Klingenburg (2008) [1]. This approach has subsequently been applied successfully to landmarking vertebral columns [2]. It uses 'partly degenerate' (or overlapping) landmarks to capture the origin of novelty in a transformational series. When the novel structure is present it is characterized by multiple distinctive landmarks. However, as it is gradually lost (or before it is gained), its absence is marked by collapsing all landmarks on the same point. In the case of the vertebral column, we use the serial homology of vertebrae to designate our landmark homologues. We advocate for this method over the alternative approach of taking separate landmark sets for each position (e.g., [3]) because it preserves the maximum information about serial variation. For example, some vertebral processes may vary not only in their degree of development but in their relative position of expression along the column, resulting in frequent 'gains' and 'losses' of the feature at a given vertebral level. However, examining variation in these structures along the column provides clear evidence for serial homology by their gradual appearance and disappearance, with their high plasticity suggesting that the potential for forming such structures is not lost but shifted antero-posteriorly [4, 5]. To exclude landmarks from these structures from some or all vertebral positions would ignore important biological information, not only about their morphology but about their positional shifts along the column. Therefore, we use information about their serial homology to guide assignments of degenerate landmarks to capture variation in these structures.

Both the metapophysis and anapophysis vary gradationally along the column, arising gradually from the lateral aspect of the prezygapophysis and the caudal arch respectively. Thus,

we capture the origin of these novel structures (both serially and between taxa) using paired landmarks as described below, with the invariant landmark of the pair underlined. The transverse process is another highly variable structure. The lumbar transverse processes (or pleuropophyses) are laterally projecting processes originating on the vertebral centrum. In the prediaphragmatic thoracic region, the diapophyseal rib facet is located on a lateral protrusion which is also known as a transverse process. In contrast, in the postdiaphragmatic thoracic region the transverse process may be completely absent, with the rib articulating directly onto the vertebra centrum. We apply a broad functional homology and categorize all lateral protrusions as transverse processes, including both pleuropophyses and diapophyses. Where projecting processes are absent, the degenerate landmark approach was used, and the landmarks were placed on the lateral margin of the vertebral arch, where the pleuropophysis emerges in the lumbar region.

Table S2: Variant landmarks

	**Partially degenerate landmarks**	
16	<u>Pre-zygapophysis - lateral extent</u>	*See above*
20	Dorsal tip of metapophysis	*Dorsal-most extent*
11	<u>Caudal arch base</u>	*See above*
21	Tip of anapophysis	*Caudal-most point*
10	<u>Cranial endplate-arch - lateral</u>	*See above*
22	Cranial tip of the transverse process	
23	Caudal tip of the transverse process	

Error study

Landmarks were collected on four taxa spanning the range of morphologies in the dataset: *Tachyglossus aculteaus* (MCZ63621, Echidna), *Didelphis virginianus* (MCZ62096, Opossum), *Felis catus* (MCZ68415, Cat), *Neotragus moschatus* (MCZ58304, Suni). The landmarking procedure was conducted on five vertebrae per species and repeated four times, totaling a sample of 20 vertebrae and 80 individual data points. Variation between repeats was assessed visually using a Principal Components Analysis on symmetrized Procrustes coordinates. The overall ability of this protocol to distinguish species and vertebrae was tested using a full-factorial MANOVA, with species and vertebra as factors, using the *procD.lm* function in the r package 'geomorph'.

Results- Repeats cluster closely in morphospace and are clearly differentiated between loci and taxa, indicating that error is sufficiently low to distinguish these morphologies (Figure S3). These visual results are confirmed by a MANOVA, which revealed highly significant differences between species and vertebrae based upon these data. The residual sums of squares of the residuals was very low, reflecting only 0.5% of the total variance in the sample. This indicates that the variance due to error was very low.

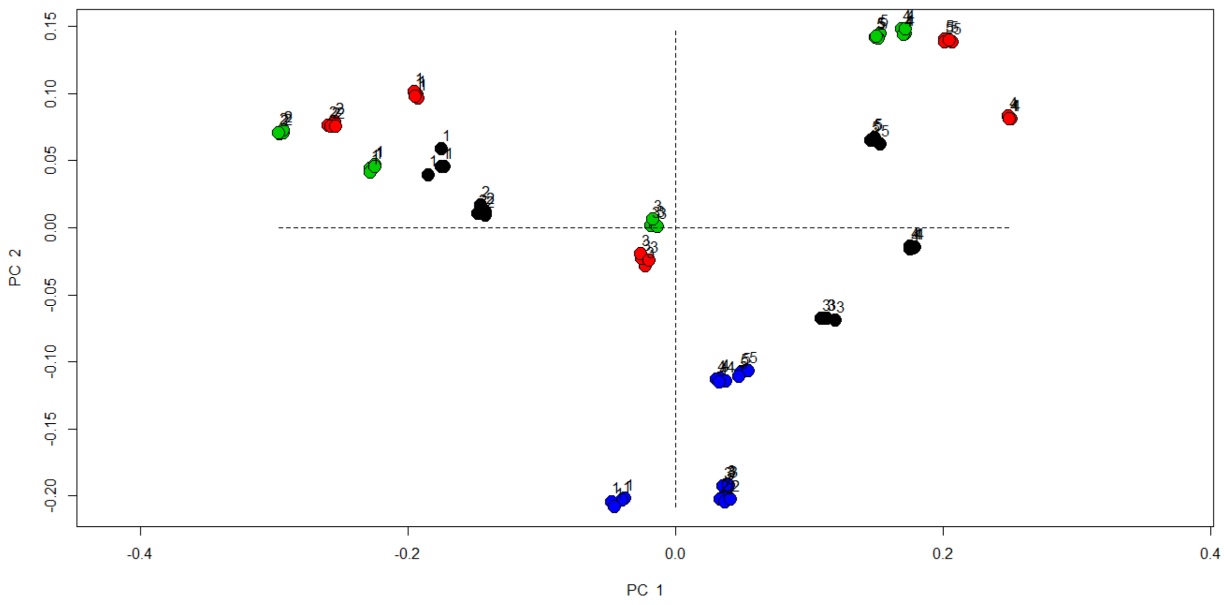

Figure S2: Variation of repeats.

Principal component analysis of all 20 vertebrae included in error study. Colors represent species; numbers indicate vertebral loci. Blue: Echidna, Black: Opossum; Red: Cat; Green: Suni.

Table S3: Error study MANOVA

	Df	*SS*	*MS*	*Rsq*	*F*	*Z*	*Pr(>F)*	
Species	*3*	*1.545*	*0.51499*	*0.22206*	*862.13*	*15.64*	*0.001*	**
Vertebra	*4*	*2.9001*	*0.72504*	*0.41684*	*1213.77*	*16.161*	*0.001*	**
Interaction	*12*	*2.4766*	*0.20638*	*0.35596*	*345.5*	*22.809*	*0.001*	**
Residuals	*60*	*0.0358*	*0.0006*					
Total	*79*	*6.9575*						

Whole-column analysis by Procrustes concatenation

To characterize evolution of the vertebral column it is necessary to consider morphological change at the level of a single vertebra, but also to consider the changes in morphological gradients along the column. Traditionally, morphometrics captures variation in a single structure based on homologous landmarks, and therefore is unable to address the variation in multiple vertebrae. Here we present a novel approach addressing the problem of the analysis of serially homologous structures using geometric morphometrics. Our approach builds on the work of Rohlf (2002) and Adams (1999), both of whom combine shape information from multiple structures by appending shape variables from a separate analyses (in their case relative warps scores) [6, 7]. Further, this approach has been applied to vertebrae by concatenating

principal components scores from individual vertebrae to examine serial variation along the column [8].

Our approach differs slightly in that we draw upon the serial homology of vertebrae to provide correspondence of structures between vertebral units, and use degenerate landmarks to capture loss or gain of serial structures (see landmarking protocol above). Therefore, we can generate identical landmark sets for each vertebral locus, allowing direct comparison of vertebrae in the same shape space. We can combine the shapes prior to ordination by concatenating Procrustes-aligned shape coordinates (‘whole column analysis’, materials and methods). In this case, where landmark sets are identical between structures, concatenating the Procrustes coordinates is *mathematically equivalent* to concatenating principal components scores as described above because the ordination is a rigid rotation of the data (see below)[9].

Simulation – We conducted a simulation study to explore the applicability of this concatenation method to gradationally varying structures. The aim of this study was to determine how well concatenated vertebral shape data could distinguish morphological gradients in shape, and the effects of differing concatenation approaches. Three base shapes were used for the simulation analysis:

Square Long rectangle Tall rectangle

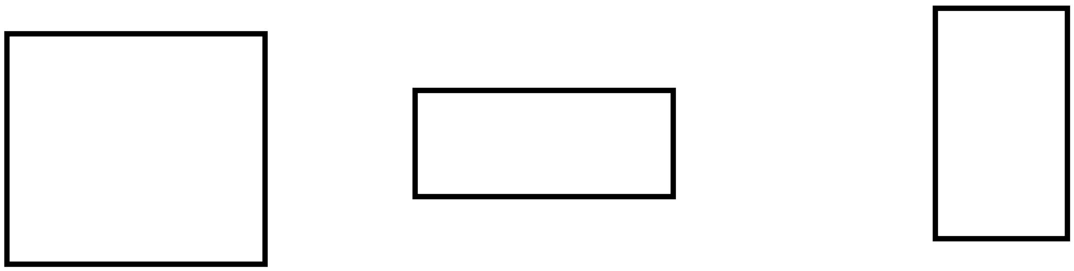

From these base shapes five hypothetical vertebral columns were created, each consisting of five shapes:

1. Square column – homogenous column consisting of five squares
2. Long column – homogenous column consisting of five long rectangles
3. Tall column – homogenous column consisting of five tall rectangles
4. Getting longer column – heterogenous column which begins as a square anteriorly then gets progressively longer
5. Getting taller column - heterogenous column which begins as a square anteriorly then gets progressively longer

From these base ‘morphotypes’, five ‘specimens’ were created for each by adding random noise. The degree of noise was set at 10%. Therefore, the analysis consists of 25 specimens of the five

morphotypes, consisting of 125 individual vertebrae. This sample of shapes was fit using Procrustes superimposition. Shape variation was analyzed using a PCA on all the vertebrae separately, and two concatenation methods: Principal Component concatenation (as in Chen et al., 2005) and Procrustes concatenation (applied here).

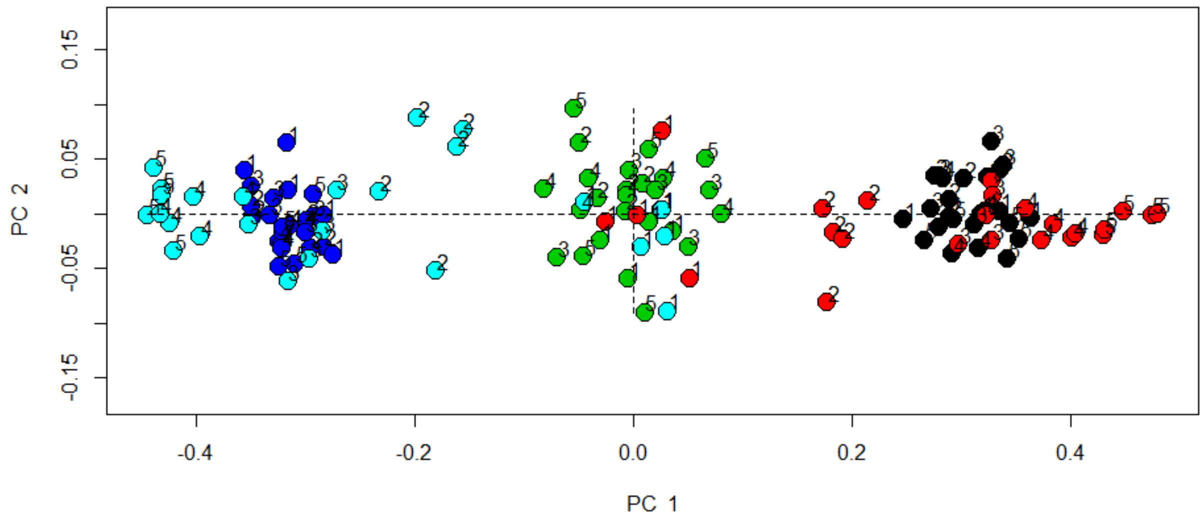

Figure S3: PCA of simulated vertebrae.
Morphotypes: Green: homogenous square; Black: homogenous long; Dark blue: homogenous tall; Red: heterogenous increasing length; Turquoise: heterogenous increasing height.

All vertebrae - Homogenous columns each occupy a single region of morphospace (dark blue=tall, green=square, black=long), whereas heterogenous columns (light blue and red) have anterior vertebrae near the squares, and increasingly disparate posterior vertebrae. Many vertebrae have overlapping shape, and the distinct morphology of each column is not detected in this traditional ‘all vertebra’ analysis because serial variation is not considered.

Concatenation- When the data are concatenated the differences between the morphotypes become much clearer (Figure S4). PC1 still distinguishes long versus tall whereas PC2 reflects the gradient along the column. This approach considers both vertebral morphology and serial gradients and therefore can distinguish the complete morphotypes. Note that the distribution in morphospace is identical irrespective of whether Procrustes or Principal Components concatenation is used. The polarity of PC2 is flipped, but the direction of PC axes is entirely arbitrary (Figure S4, S5). This is demonstrated by the perfect correlation of PC scores resulting from the two methods (Table S4).

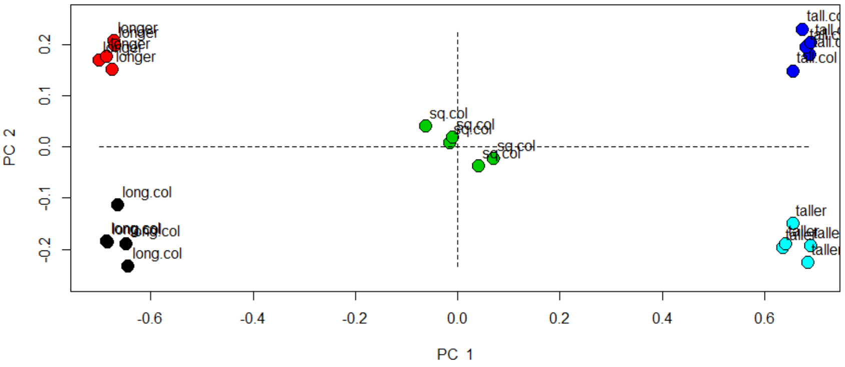

Figure S4: Procrustes concatenation of simulation data

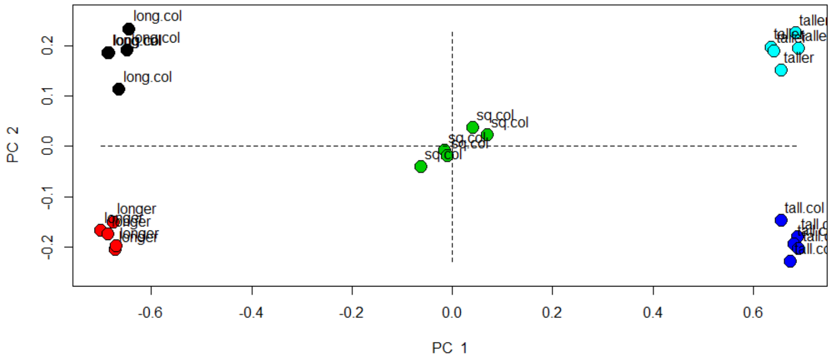

Figure S5: Principal Component concatenation of simulation data

PC1	1	PC11	-1
PC2	-1	PC12	-1
PC3	-1	PC13	-1
PC4	-1	PC14	-1
PC5	1	PC15	-1
PC6	1	PC16	1
PC7	1	PC17	-1
PC8	1	PC18	1
PC9	1	PC19	-1
PC10	-1		

Table S4: Correlation between PC scores generated by Procrustes and Principal Component concatenation.

ADDITIONAL TABLES

Table S5: Taxonomic sample.
Locomotor categories are defined as in [10]. Secondary classifiers are indicated in parentheses.

SA: Scansorial-arboreal; T: Terrestrial; Aq: Semi-aquatic; F: Fossorial.

Species	Common name	Sp. No	Ecology	Ecology Reference
Ailurus fulgens	Red panda	MCZ52237	SA	Fabre, 2015
Alouatta palliata	Mantled howler monkey	MCZ 47267	SA	Nowak, 1999
Antilocapra americana	Pronghorn	MCZ BOM-1773	T	Nowak, 1999
Arctidis binturong	Biturong	MCZ35594	SA	Samuels et al., 2013
Callorhinus ursinus	Northern fur seal	MCZ1787	Aq	Nowak, 1999
Caluromys philander	Bare-tailed woolly opossum	MCZ32359	SA	Argot, 2003
Castor canadensis	American beaver	MCZ 64159	Aq	Samuels and Van Valkenburgh, 2008
Choloepus hoffmani	Hoffmann's two-toed sloth	MCZ 12348	SA	White, 1993
Chrysochloris stuhlmanni	Stuhlmann's golden mole	AMNH82372	F	Nowak, 1999
Crocuta crocuta	Spotted hyena	MCZ 20968	T	Samuels et al., 2013
Cuniculus paca	Lowland paca	MCZ BOM 829	T	Nowak, 1999
Dasypus novemcinctus	Nine-banded armadillo	SEP1	F	Samuels and Van Valkenburgh, 2008
Dendrohyrax dorsalis	Western tree hyrax	MCZ 6069	SA	Nowak, 1999
Didelphis viginiana	Virginia opossum	MCZ 62096	SA(T)	Chen and Wilson, 2015; Argot, 2001; Kirk et al., 2008
Equus caballus	Horse	MCZ 14915	T	Nowak, 1999
Erethizon dorsatum	North American porcupine	MCZ BOM 965	SA	Samuels and Van Valkenburgh, 2008
Erinaceus europaeus	European hedgehog	MCZ6021	T (SA)	Nowak, 1999
Felis catus	Housecat	MCZ 68415	T (SA)	Nowak, 1999
Gorilla gorilla	Gorilla	MCZ29048	SA	Nowak, 1999
Hemicentetes semispinosus	Lowland streaked tenrec	AMNH100837	F	Chen and Wilson, 2015
Hydrochoerus hydrochaeris	Capybara	MCZ BOM 6013	Aq (T)	Samuels and Van Valkenburgh, 2008
Lama guanaco	Guanaco	MCZ BOM - 1881	T	Nowak, 1999

Lepus americanus	Snowshoe hare	MCZ 852	T	Seckel and Janis, 2008
Lutra lutra	European otter	UMCZ K2768	Aq	Samuels et al., 2013
Lycaon pictus	African wild dog	MCZ 13233	T	Samuels et al., 2013
Macropus robustus	Common wallaroo	MCZ 63609	T	Pfaff et al., 2017
Manis Temminckii	Ground pangolin	MCZ 34184	F (SA)	Nowak, 1999
Marmota monax	Groundhog	MCZ BOM 377	F (T)	Van Valkenburgh, 1987; Kirk et al., 2008
Mus musculus	House mouse	MCZ 59560	T (F)	Nowak, 1999
Myrmecophaga tridactyla	Giant anteater	MCZ 20969	F	White, 1993
Nectomys squamipes	South American water rat	MCZ37898	Aq	Samuels and Van Valkenburgh, 2008
Neotragus moschatus	Suni	MCZ 58304	T	Nowak, 1999
Neovision neovision	American mink	MCZ47131	Aq	Samuels and Van Valkenburgh, 2008
Odocoileus virginianus	White-tailed deer	MCZ 46590	T	Nowak, 1999
Orycteropus afer	Aadvark	MCZ 20970	F	MacLeod and Rose, 1993
Ovis aries	Sheep	MCZ BOM 6338	T	Nowak, 1999
Phascolarctos cinereus	Koala	MCZ 58136	SA	Pfaff et al., 2017
Potamogale velox	Giant otter shrew	MCZ38059	Aq	Nowak, 1999
Procyon lotor	Racoon	MCZ 7101	SA (T)	Samuels et al., 2013; Kirk et al., 2008
Sciurus carolinensis	Eastern gray squirrel	MCZ61742	SA	Chen and Wilson, 2015
Solenodon paradoxus	Hispaniolan solenodon	MCZ12381	F	Chen and Wilson, 2015
Sus scrofa	Wild boar	MCZ BOM-6246	T	MacLeod and Rose, 1993
Tachyglossus aculeatus	Short-beaked echidna	MCZ 63621	F	Clemente et al., 2016
Talpa Europaea	European mole	MCZ2353	F	Nowak, 1999
Tamandua tetradactyla	Southern tamandua	MCZ 20965	F	White, 1993
Tapirus bairdii	Baird's tapir	MCZ BOM-1076	T	MacLeod and Rose, 1993
Tupaia minor	Pygmy treeshrew	FMNH6865	SA	Chen and Wilson, 2015
Tupaia palawanensis	Palawan treeshrew	FMNH62976	SA	Nowak, 1999
Ursus americanus	American black bear	MCZ59938	T	Nowak, 1999
Varecia veregata	Black-and-white ruffed lemur	MCZ 18740	SA	Kirk et al., 2008
Vombatus ursinus	Common wombat	MCZ 24974	F	Pfaff et al., 2017

Zaglossus bruijni	Western long-beaked echidna	MCZ 12414	F	Clemente et al., 2016

ADDITIONAL FIGURES

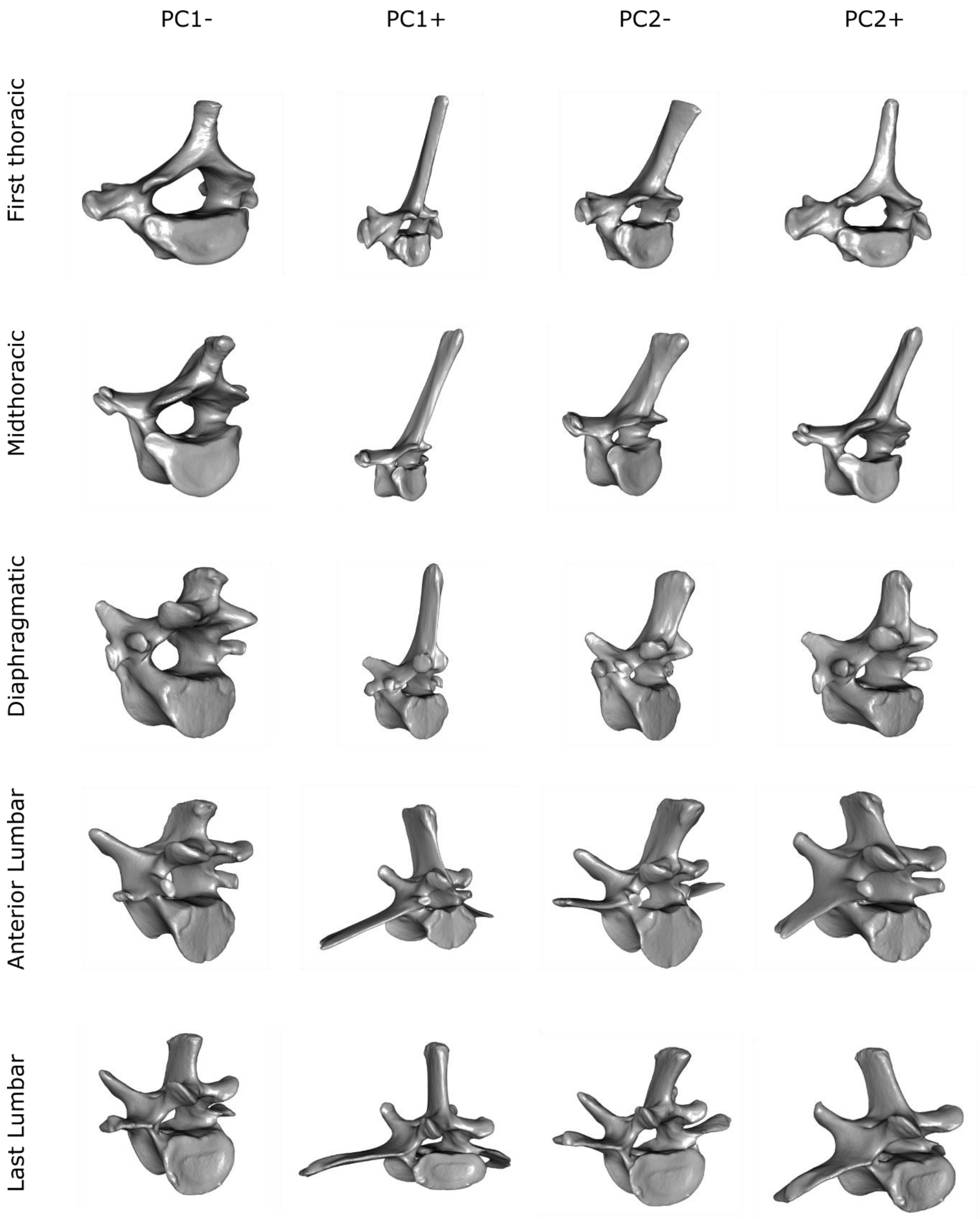

Figure S6: Shape variation associated with whole-column concatenated PCA visualized by mesh warping.

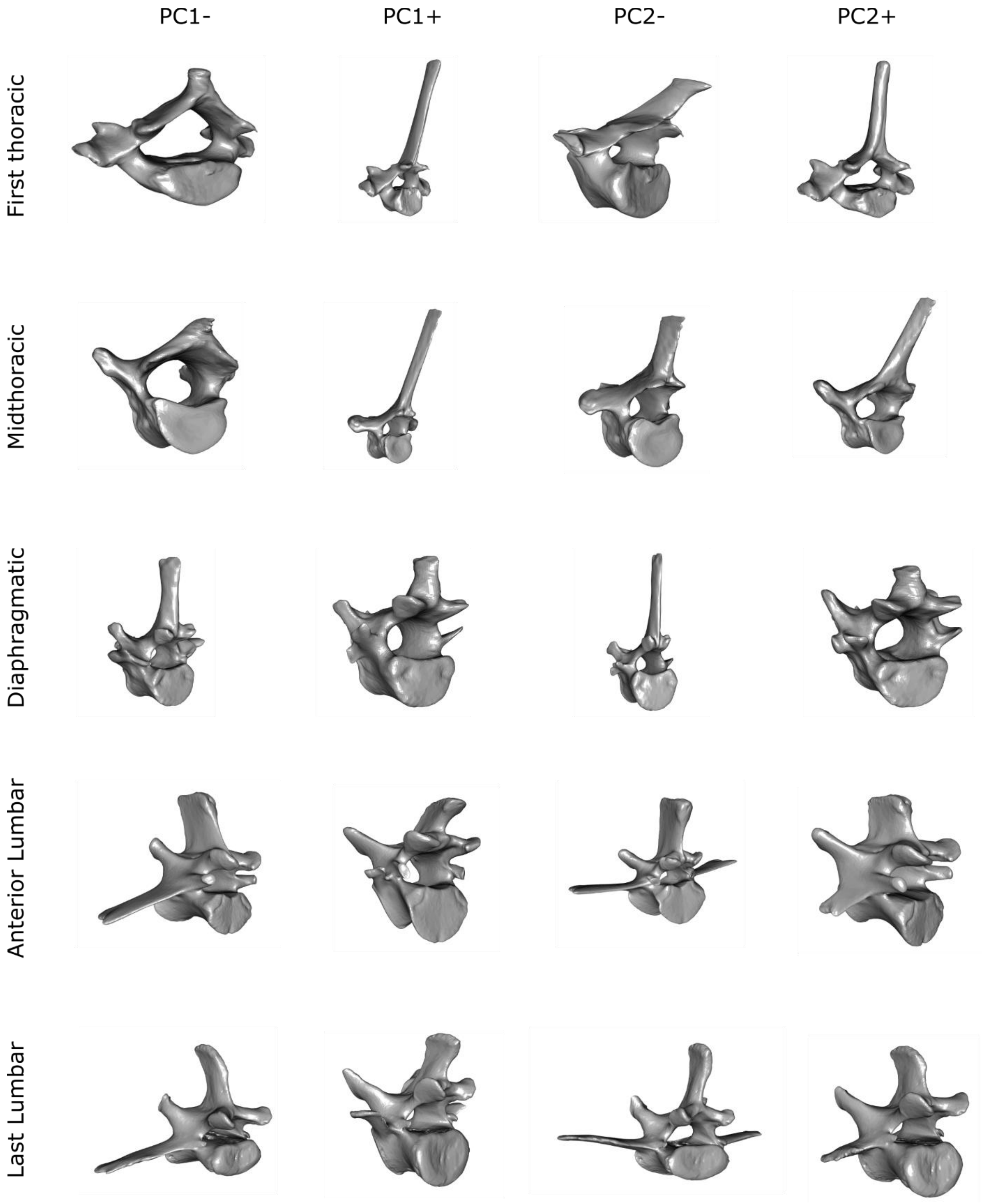

Figure S7: Shape variation associated with individual PCAs visualized by mesh warping.

SUPPLEMENTARY REFERENCES

1. Klingenberg CP: **Novelty and “homology-free” morphometrics: what’s in a name?** *Evol Biol* 2008, **35**(3):186-190.
2. Head JJ, Polly PD: **Evolution of the snake body form reveals homoplasy in amniote Hox gene function**. *Nature* 2015, **520**(7545):86-89.
3. Randau M, Cuff AR, Hutchinson JR, Pierce SE, Goswami A: **Regional differentiation of felid vertebral column evolution: a study of 3D shape trajectories**. *Org Divers Evol* 2017, **17**(1):305-319.
4. Filler AG: **Homeotic evolution in the Mammalia: diversification of therian axial seriation and the morphogenetic basis of human origins**. *PLoS ONE* 2007, **2**(10):e1019.
5. Buchholtz E: **Flexibility and constraint: patterning the axial skeleton in mammals**. *From Clone to Bone: The Synergy of Morphological and Molecular Tools in Palaeobiology* 2012, **4**:230.
6. Rohlf FJ: **Geometric morphometrics and phylogeny**. *Morphology, shape and phylogeny* 2002:175-193.
7. Adams DC: **Methods for shape analysis of landmark data from articulated structures**. *Evol Ecol Res* 1999, **1**(8):959-970.
8. Chen XM, Milne N, O'Higgins P: **Morphological variation of the thoracolumbar vertebrae in Macropodidae and its functional relevance**. *J Morphol* 2005, **266**(2):167-181.
9. Zelditch M, Swiderski D, Sheets HD, Fink W: **Geometric Morphometrics for Biologists: A Primer**. Boston, MA: Elsevier Academic Press; 2004.
10. Samuels JX, Meachen JA, Sakai SA: **Postcranial morphology and the locomotor habits of living and extinct carnivorans**. *J Morphol* 2013, **274**(2):121-146.
11. Fabre AC, Cornette R, Goswami A, Peigné S: **Do constraints associated with the locomotor habitat drive the evolution of forelimb shape? A case study in musteloid carnivorans**. *J Anat* 2015, **226**(6):596-610.
12. Nowak RM: **Walker's mammals of the world**, vol. 1: JHU Press; 1999.
13. Argot C: **Functional-adaptive anatomy of the axial skeleton of some extant marsupials and the paleobiology of the Paleocene marsupials *Mayulestes ferox* and *Pucadelphys andinus***. *J Morphol* 2003, **255**(3):279-300.
14. Samuels JX, Van Valkenburgh B: **Skeletal indicators of locomotor adaptations in living and extinct rodents**. *J Morphol* 2008, **269**(11):1387-1411.
15. White JL: **Indicators of locomotor habits in xenarthrans: evidence for locomotor heterogeneity among fossil sloths**. *J Vert Paleontol* 1993, **13**(2):230-242.
16. Chen M, Wilson GP: **A multivariate approach to infer locomotor modes in Mesozoic mammals**. *Paleobiology* 2015, **41**(2):280-312.
17. Kirk EC, Lemelin P, Hamrick MW, Boyer DM, Bloch JI: **Intrinsic hand proportions of euarchontans and other mammals: implications for the locomotor behavior of plesiadapiforms**. *J Hum Evol* 2008, **55**(2):278-299.
18. Seckel L, Janis C: **Convergences in scapula morphology among small cursorial mammals: an osteological correlate for locomotory specialization**. *J Mamm Evol* 2008, **15**(4):261.
19. Pfaff C, Czerny S, Nagel D, Kriwet J: **Functional morphological adaptations of the bony labyrinth in marsupials (Mammalia, Theria)**. *J Morphol* 2017, **278**(6):742-749.
20. Van Valkenburgh B: **Skeletal indicators of locomotor behavior in living and extinct carnivores**. *J Vert Paleontol* 1987, **7**(2):162-182.

21. MacLeod N, Rose KD: **Inferring locomotor behavior in Paleogene mammals via eigenshape analysis**. *Am J Sci* 1993, **293**(A):300.
22. Clemente CJ, Cooper CE, Withers PC, Freakley C, Singh S, Terrill P: **The private life of echidnas: using accelerometry and GPS to examine field biomechanics and assess the ecological impact of a widespread, semi-fossorial monotreme**. *J Exp Biol* 2016, **219**(20):3271-3283.
